# Addressing Racial Compatibility Between Trainees and Minority Older Adults in Applied Care Settings

**DOI:** 10.1093/geroni/igaa057.3055

**Published:** 2020-12-16

**Authors:** Danielle McDuffie

**Affiliations:** The University of Alabama, Tuscaloosa, Alabama, United States

## Abstract

This talk explores the importance of a racially diverse graduate student interacting in an applied care setting with minority older adults in the Southeast relative to assessment of cognitive status and in the context of cultural mistrust and misdiagnosis. ~250 older adults (16% AA) in an applied care setting were administered a cognitive screener as part of a larger research battery. An independent samples t-test was conducted to assess differences in mean cognitive status. Non-Hispanic Whites (NHW) (M=76.8 years) were found to be more intact than AAs (M=73.27) on measures of cognitive status (21.32 vs. 14.72, t=4.976, p=.0001). Implications highlight that cognitive screeners have often been found to lack sensitivity in groups of marginalized older adults. Having an AA graduate student in these settings could be a way of mitigating the effects of culturally incompatible screening tools and bridging the gap between research and practice for AA older adults.

